# NMPhenogen: a comprehensive database for genotype–phenotype correlation in neuromuscular genetic disorders

**DOI:** 10.3389/fnins.2025.1696899

**Published:** 2025-11-04

**Authors:** Usha Manjunath, Venkatesh R, Sacheta Sudhendra Kulkarni, Harshatha N. Reddy, Anupama Anil, Rakesh Kumar Mishra, Gayatri Rangarajan Iyer

**Affiliations:** Tata Institute for Genetics and Society, Gandhi Krishi Vignana Kendra Campus, Bengaluru, Karnataka, India

**Keywords:** neuromuscular disorders, NMGD, NMPhenoscore, variant classifier, variant interpretation tool, disease prioritization, ACMG, variant classification

## Abstract

Neuromuscular genetic disorders (NMGDs) are genetically and clinically diverse group of inherited diseases that affect approximately 1 in 1,000 people worldwide with a calculated prevalence of 37 per 10,000 in the general population. These disorders arise from a variety of genetic changes such as insertions, deletions, duplications and expansions of repeats in more than 747 nuclear and mitochondrial genes critical for the function of peripheral nerves, motor neurons, neuromuscular junctions or skeletal muscles, leading to progressive weakness and degeneration of muscles. Major subtypes include muscular dystrophies, congenital myopathies, motor neuron diseases, peripheral neuropathies, and mitochondrial myopathies. Clinical presentation of NMGDs is highly variable in the age of onset, severity and pattern of muscle involvement, often leading to prolonged and complex diagnostic process. Conventional diagnostic methods have relied on clinical history, physical examination and invasive procedures like muscle biopsy. But the development of next-generation sequencing (NGS) has transformed diagnostics by enabling comprehensive analysis of NMGD-related genes. Despite this advancement, interpreting the numerous variants identified by NGS remains challenging. The guidelines of the American College of Medical Genetics and Genomics (ACMG) offer a standardized approach to variant classification as pathogenic, likely pathogenic, variant of uncertain significance, likely benign and benign. However, this requires the integration of complex evidence from population data, computational predictions, and functional assays. The major challenge is the robust correlation of genotypic information with the huge phenotypic range of NMGDs which is a task complicated by the unavailability of population-specific genetic databases. To address these issues, we have developed NMPhenogen (https://gi-lab-tigs.github.io/Homepage/), a new database designed to enhance the diagnosis and understanding of NMGDs. NMPhenogen is a centralized repository for data related to NMGD-associated genes and variants along with their clinical presentations. It includes two primary modules: NMPhenoscore, which enhances disease-phenotype correlations, and a Variant classifier, which facilitates standardized variant classification based on published guidelines. This combined resource aims to streamline the diagnostic process, support clinical decision-making, and eventually contribute to improving patient care and genetic counseling.

## Introduction

Neuromuscular genetic disorders (NMGDs) are a heterogeneous group of genetic disorders that affect around 1 in 1,000 individuals worldwide (Zatz et al., 2016). Although each condition is rare, they are collectively common with an overall prevalence estimated to be 37 per 10,000 in the general population (Rosenberg et al., 2023). NMGDs result from genetic variants including insertions, deletions, duplications, or repeat expansions that disrupt the function of peripheral nerves, motor neurons, neuromuscular junctions, or skeletal muscles, leading to progressive muscle weakness and degeneration (Zatz et al., 2016; Rosenberg et al., 2023; Laing, 2012). There are around 747 different genes including both nuclear and mitochondrial genes, resulting in 1240 NMGDs (Benarroch et al., 2025; Baig et al., 2019). These genes code for proteins that are involved in muscle structure and function (*DMD*, *SGCA*, *LMNA*), neuromuscular junction transmission (*CHRNE, RAPSYN*), mitochondrial energy metabolism (*POLG, TK2*), peripheral nerve functions (*PMP22, MFN2, GJB1*) etc. NMGDs are mostly monogenic, inherited as autosomal dominant (Myotonic dystrophy, LGMD1A-1H), autosomal recessive (LGMD 2A-2 T), X-linked (DMD), mitochondrial (MERRF, MELAS) or can be *de novo* (Nemaline Myopathy- *ACTA1* gene) (Zatz et al., 2016; Laing, 2012; Arakawa et al., 2018; Malfatti and Romero, 2016). Major subtypes of NMGDs include-

1. Muscle dystrophies: Duchenne muscular dystrophy (most common muscular dystrophy with childhood onset), Becker muscular dystrophy, Limb girdle muscular dystrophy, congenital muscular dystrophy, Facioscapulohumeral muscular dystrophy (FSHD), Emery-Dreifuss muscular dystrophy, Oculopharyngeal muscular dystrophy (OPMD), Myotonic dystrophy (DM1 being the most common muscular dystrophy with adult onset and the estimated prevalence of about 1:20,000) (Bird, 2019).
2. Congenital myopathies: Nemaline myopathy
3. Motor neuron disorders: 5q spinal muscular atrophy, Amyotrophic lateral sclerosis
4. Hereditary peripheral neuropathies: Charcot–Marie–Tooth disease, Hereditary motor neuropathies, Hereditary sensory and autonomic neuropathies
5. Neuromuscular junction disorders: Congenital myasthenic syndromes
6. Metabolic myopathies: Glycogen storage disorders, Fatty acid oxidation disorders
7. Mitochondrial myopathies: Leigh syndrome (infant/childhood onset), Mitochondrial encephalopathy lactic acidosis and stroke like episodes (MELAS), Myoclonic epilepsy with ragged red fibers (MERRF) (childhood/adult onset), Chronic Progressive External Ophthalmoplegia (CPEO) (representing 20% of adult onset mitochondrial disorders) (Arakawa et al., 2018; Mary et al., 2018; Chin et al., 2024).

The onset, severity and clinical presentation of NMGDs vary widely. Disorders, such as congenital myopathies and congenital muscular dystrophies (CMDs), manifest at birth or in early infancy with hypotonia, delayed milestones, or respiratory insufficiency, while others, such as Limb girdle muscular dystrophy (LGMD) or Facioscapulohumeral muscular dystrophy (FSHD), present during adolescence or adulthood (Arakawa et al., 2018). Duchenne muscular dystrophy (DMD), the most prevalent and severe form affects approximately 1 in 3,500 male children and typically presents before the age of five with progressive proximal muscle weakness, calf hypertrophy, and elevated creatinine phosphokinase (CPK) levels (Kashyap et al., 2019). CMDs, though less common (0.6–0.9 per 100,000), present with central nervous system (CNS), ocular and cardiac involvement (Sindhav et al., 2025). Mitochondrial disorders affect multiple systems with a wide variety of neurological, muscular, hepatic, and gastrointestinal symptoms. Mitochondrial myopathies, with the estimated prevalence of 1 in 5,000, are linked to defects in oxidative phosphorylation, presenting with exercise intolerance, cramps, fatigue, myalgias, or metabolic decompensation during illness or fasting. Individuals with childhood onset have more generalized muscle and systemic involvement while those with adult onset have milder phenotypes confined to specific muscles (Chin et al., 2024; Ahmed et al., 2018). Similarly, hereditary spastic paraplegias (HSPs) occur in about 1–9 per 100,000 individuals and are characterized by progressive spastic paraplegias due to distal axonal degeneration (Arakawa et al., 2018; Mahesan et al., 2024). Despite this diversity, muscle weakness- whether proximal, distal, axial, facial, or respiratory- remains the unifying clinical hallmark of NMGDs. Patterns of weakness, in combination with examination findings, provide crucial diagnostic clues. Waddling gait, for instance, is indicative of the proximal and axial involvement majorly seen in LGMD, while steppage or foot drop denotes distal weakness, which is present in inclusion body myositis, distal myopathies, or myotonic dystrophy (Dubrovsky, 2018). Peculiar clinical signs, such as calf hypertrophy in DMD and sarcoglycanopathies, ‘lumpy-bumpy’ quadriceps in dysferlinopathy, scapular winging in LGMD, facial weakness with scapulo-peroneal distribution in FSHD, and tongue fasciculations in SMA are valuable in narrowing the diagnostic possibilities (Arakawa et al., 2018; Dubrovsky, 2018). Systemic involvement also aids recognition and early intervention, for example cardiac manifestations in DMD and myotonic dystrophy, hepatomegaly in metabolic storage disorders, early respiratory failure in nemaline myopathy, and contractures in Emery-Dreifuss muscular dystrophy (Mahesan et al., 2024).

Traditionally, diagnosis of NMGDs has relied on clinical history, neuromuscular examination, family pedigree analysis, and invasive investigations such as muscle biopsy for dystrophies, myopathy and to assess nervous involvement, electromyography (EMG) for categorizing myopathies, Magnetic Resonance Imaging (MRI) for muscle involvement patterns and targeted enzyme assays such as acid maltase testing for Pompe Disease. NMGDs present with non-specific symptoms such as hypotonia, weak cry, poor feeding, or respiratory distress which might be mistaken with other prenatal disorders. Disorders like myotonic dystrophy caused due to repeat expansion and FSHD, involving 3.2 kb contraction as well as methylation complicates the diagnosis since non-conventional tests are required, further delaying the diagnosis. Families frequently endure a prolonged diagnostic odyssey due to overlapping clinical symptom, genetic heterogeneity, lack of awareness and access to diagnostics that includes multiple tests, consultations, invasive procedures before reaching a molecular diagnosis. This can result in missed treatment opportunities and preventable mortality (Rosenberg et al., 2023). Patients experience pain, exhaustion, low mood, poor coping, and increased unfavorable perceptions of their illness in addition to physical disability (Graham et al., 2011). About 34% of all the childhood deaths, and over 51% of those under one year of age, are attributed to congenital malformations or genetic disorders, including severe NMGDs such as fetal akinesia, congenital myopathies and CMDs (Stevenson and Carey, 2004). As the conditions are heritable in nature, often times they recur in the same family. Delayed molecular diagnosis also poses a barrier for timely and informed reproductive planning. The missed window can lead to recurrence and add to the burden of neonatal morbidity and mortality of NMGDs. The advent of next generation sequencing (NGS), particularly whole exome sequencing, has revolutionized the diagnostic picture of NMGDs by enabling rapid, comprehensive identification of causative variants. Variant analysis and reporting are significant to understand the etiology of the disorder, nature of progression, employ targeted therapy if any, or facilitate early intervention and aid in prevention in subsequent pregnancies by prenatal diagnosis and extended family screening. Variant analysis, however, is often not straightforward and has several layered steps of annotating sequence data, analyzing population frequencies, *in silico* computational predictions, *in vitro* and model system generated functional assays, segregation analysis, thorough genotype–phenotype correlation and ranking of variants accordingly. Lack of population specific databases can hinder optimum utility of sequence data as several ethnicity and community specific variants remain underrecognized and unknown.

The American College of Medical Genetics (ACMG) guidelines are the standard recommendations for the interpretation and reporting the sequence variations identified to provide an educational resource for medical geneticists and other health care professionals to provide quality medical genetic services (Richards et al., 2015). It provides a framework for interpretation and reporting of the test results and to aid clinicians by educating them to possible testing outcomes so that they can inform their patients and families appropriately (Richards et al., 2015; Richards et al., 2008). A workgroup was established in 2013 by ACMG, Association for Molecular Pathology (AMP), and College of American Pathologists (CAP) with the objective of creating a recommendation for the use of standard terminologies for categorizing sequence variants based on the evidence that is currently available and weighted in accordance with a system that was created using community input, workgroup consensus, and expert opinion. These recommendations involve the five classes namely pathogenic (P), likely pathogenic (LP), benign (B), likely benign (LB) and variant of uncertain significance (VUS). Each classification is based on the studies done and supporting evidence available, which describe each variant with respect to different characteristics and degrees of pathogenicity (Richards et al., 2015; Nicora et al., 2022). Pathogenic criterion is scored as very strong with one criterion (PVS1), four strong parameters (PS1-4), six moderate parameters (PM1-6), and about five supporting parameters (PP1-5), Benign criterion as standalone (BA1), about four strong parameters (BS1-4), and seven supporting parameters (BP1-7). According to ACMG guidelines 2015, the classification requires evidence ranging from molecular and population data to clinical and familial correlations. One needs to consider the variants effect on the protein activity whether it is a nonsense, frameshift, splice site or missense leading to loss of function, if the variant is present in the conserved sites as substitutions at these sites are often pathogenic, if the variant allele frequency in the population is <1%, segregation analysis with the family also provides strong evidence with the disease if present in multiple affected family members. Genotype–Phenotype correlation plays a major role in explaining the clinical features observed, inheritance pattern (eg. Hemizygous mutation in *DMD* gene explains affected males and carrier females), allelic heterogeneity (missense variants in LMNA gene cause cardiomyopathy while truncating variants cause muscular dystrophy), Each of this evidence contribute to different strengths (very strong, strong, moderate and supporting) resulting in the final classification. Evidence for a particular variant from each criterion is combined to classify it into one of the five classes (Richards et al., 2015). It is important to make sure that the variants are reported precisely, especially while reporting pathogenic and likely pathogenic variants, as it can lead to alterations in the treatment or surveillance of the patient. As the research field grows functional and clinical validations are carried out enabling more evidence, variant reclassification becomes indispensable. With the advancement of NGS technologies, thousands of variants are identified for an individual, of these only few might be disease causing. However, VUS may have a conflicting interpretation due to lack of information. In such scenarios, clinical geneticists and clinical-laboratory geneticists healthcare providers depend on *in silico* tools that assess protein variant tolerance, splicing or gene regulatory alterations by assigning quantitative measures (Nicora et al., 2022). ClinVar^1^ is a freely accessible, public archive of reports of human variations classified for diseases and facilitates access to human variation and asserted relationships observed conditions with interdatabase operability. Currently, tools like Varsome,^2^ Franklin,^3^ LOVD^4^ InterVar^5^ and GeneBe^6^ are available for variant interpretation based on ACMG guidelines.

We have developed a database—NMPhenogen, to aid and improve genotype–phenotype correlation in neuromuscular genetic disorders. It incorporates the NMPhenoscore feature which assesses the likelihood of NMGDs based on the presence of 24 clinically relevant features used as a clinical domain (Kashyap et al., 2019; Mancuso et al., 2025; Mcdonald, 2012; Semplicini and Angelini, 2013; Version, 2002) during the initial screening phase and then gives the top recommendations for probable NMGDs diagnosis based on the relevant clinical data provided by the user. The database also assist in variant classification according to ACMG guidelines using the variant calculator feature. Additionally, the database provides information about different genes and their inheritance patterns associated with neuromuscular disorder subtypes along with their clinical features.

### Objectives

To develop an integrated platform for genotype–phenotype spectrum and variant classification for neuromuscular genetic disorders.

1. NMPhenoscore: To assist clinicians, genetic counselors, laboratory and clinical scientists in genotype–phenotype correlation and selecting gene panels, as well as
2. Variant classifier: A tool to classify variants in a user friendly intuitive prompt based format according to ACMG guidelines.

## Methodology

**Figure 1:**
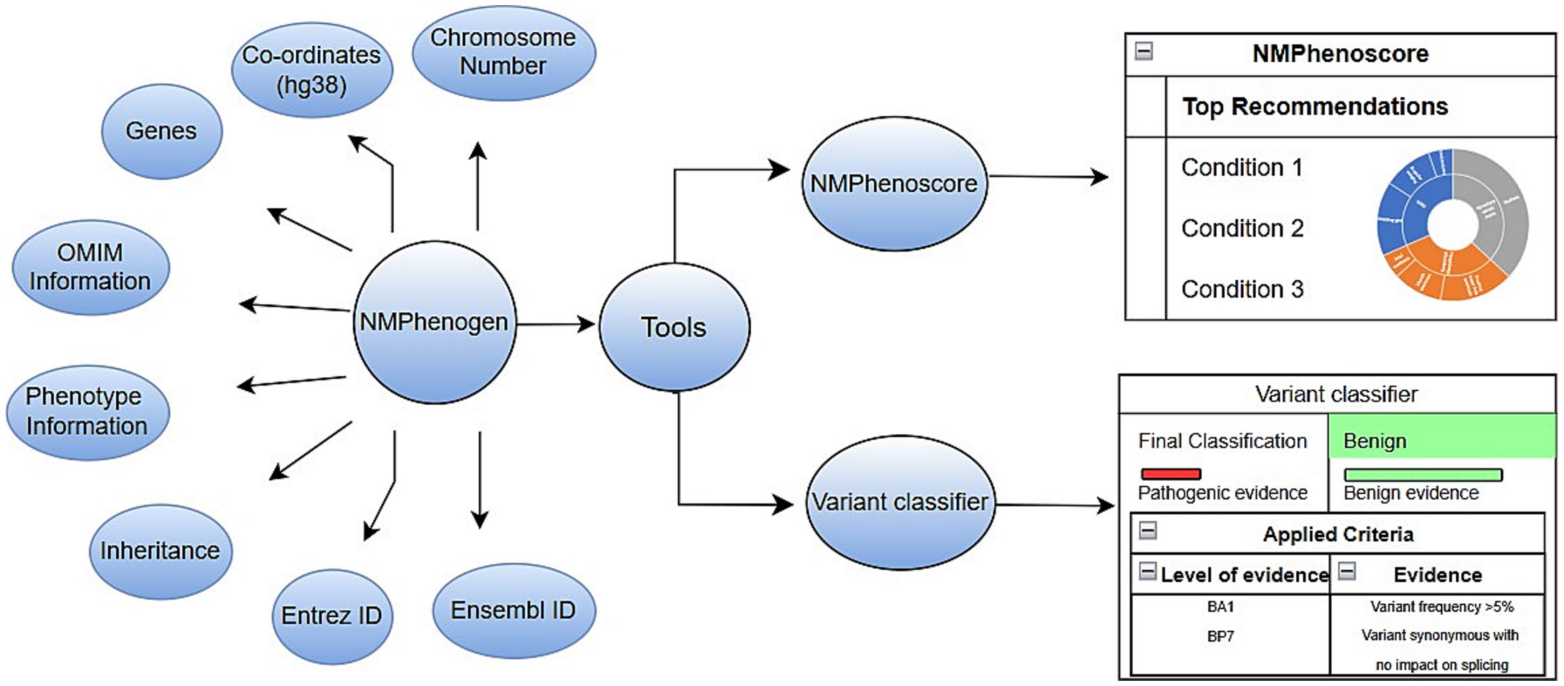
Overview of the database.

We have developed NMPhenogen database using JavaScript, which is the collection of 747 genes that are associated with neuro muscular genetic disorders (Supplementary file 1). These 747 genes, including the ones from mitochondrial genome were collected from various literature and publicly available NMGD databases namely GeneTable and NeuroMuscleDB (Benarroch et al., 2025; Baig et al., 2019). This database contains the comprehensive information of these genes which includes chromosome number, position, associated diseases, inheritance pattern, along with Online Mendelian Inheritance in Man (OMIM)^7^ and phenotype links related to Mendelian Inheritance in Man (MIM) (see the text footnote 7), Orphanet,^8^ MalaCards,^9^ Entrez Gene Identifier (Entrez ID)^10^ and links Ensembl Gene Identifier (Ensembl ID).^11^ Inheritance pattern and disease information were collected from OMIM along with the corresponding OMIM and phenotype MIM links. The database includes NMPhenoscore a tool to prioritize NMGDs based on the clinical features given by the users, and Variant classifier, a variant interpretation tool which classifies variants based on the ACMG guidelines. Figure 1 summarizes the overall picture of the database.

### The NMPhenoscore

NMPhenoscore is a web-based tool that prioritizes neuromuscular genetic disorders related to medical conditions based on their clinical symptoms. NMPhenoscore functions in two parts. First part is where users input symptoms that are considered to be the broad clinical domains affected for evaluating the patients. Second part of the tool involves predicting most likely specific conditions of NMGDs. The main purpose of this tool is to serve as a clinical decision support system (CDSS). It helps the medical professionals, geneticists, and clinicians in diagnosing rare NMGDs by comparing the patients’ symptoms to a well-structured knowledgebase to narrow down the large number of differential diagnoses for these rare diseases, improving the efficacy of the diagnostic process. This cuts down the time of the initial diagnostic stage and offers a methodical, fact-based foundation for additional research. Additionally, it helps clinical researchers find patient cohorts for studies and acts as an educational aid for medical students learning about rare diseases.

The tool is developed using JavaScript and HTML. It is structured into three core phases such as data acquisition and preprocessing, algorithm design and user interaction and output generation.

**Figure 2:**
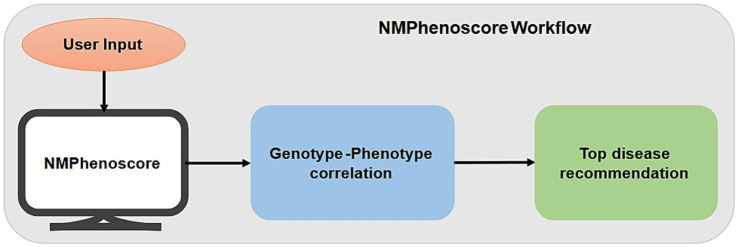
Workflow of NMPhenoscore tool.

- *Data acquisition and preprocessing*: The first part of the NMPhenoscore tool involves evaluating patients for potential NMGDs using approximately 24 clinical symptoms that were curated through a comprehensive review of the literature (listed in Supplementary file 2-sheet 2). The foundation for the second part of the tool is a structured knowledgebase of disease-symptoms which were curated manually from the literature available. This knowledgebase contains 141 symptoms distributed among 34 clinical conditions (given in Supplementary Figure 1 and the data in Supplementary file 2). A data frame is developed where rows represent symptoms and columns represent medical conditions related to NMGD. Within this data frame, the presence of prominent association between symptom and condition is represented as ‘+’ whereas the absence or rarity of the association is represented as empty cells. To make the data computationally operable, two tasks have been performed. First, indexing has been done for the first column, which contains symptoms, to create a structured lookup table. Second is the binary encoding where all the ‘+’ symbols are replaced with 1 and empty cells with 0.
- *Algorithm design*: The first part of the tool aggregates 24 binary clinical indicators, with each symptom representing equally to 1 point. The algorithm calculates a percentage score by dividing the number of selected symptoms by the total possible symptoms and multiplying by 100. A critical diagnostic threshold is established at 30% scores at or above this threshold to classify the patient as “NMGD likely Positive,” while scores below 30% are classified as “NMGD likely Negative.” The visual interface employs a circular gauge that dynamically updates to reflect the calculated percentage, with color-coding which has green for positive, amber for negative providing immediate visual feedback. The core of the second part of the tool is a prioritization algorithm that is based on a simple and yet effective scoring method which quantifies the presence of symptoms and the conditions in the data frame. The tool accepts a user-provided list of symptoms and validates it against the index of the symptoms present in the preprocessed data frame. Validated symptoms are retained and filtered, whereas unrecognized ones are excluded for further processing. The data frame now contains the validated symptoms to which a conditional score is calculated for each disease by summing the binary values (1 and 0) across all provided symptoms. The score effectively represents the total number of symptoms the user provided that are known to be associated with each condition. The conditions are then ranked in descending order based on their calculated scores. This results in a prioritized list of conditions with the maximum number of matching symptoms getting the highest rank.
- *User Interaction and Output Generation:* The tool features the user-friendly interface where the user is prompted to enter the list of symptoms one by one. The output gets generated in two parts. One part gives the list of the conditions along with the number of matched symptoms in descending order. The second part gives the top recommended condition with the absolute highest score. Figure 2 depicts the workflow of NMPhenoscore tool.

### Variant classifier

Variant classifier is a user-friendly interface designed to assist clinicians, researchers, geneticists, genetic counsellors, and molecular laboratories to categorize genetic sequence variants according to the widely adopted the American College of Medical Genetics and Genomics (ACMG) and the Association for Molecular Pathology (AMP) guidelines. With the increase in gene testing and discovery of new variants, there is a critical need for an open, standardized, and user-friendly system to support variant interpretation. The tool uses the five-tier classification system namely pathogenic, likely pathogenic, variant of uncertain significance, likely benign, and benign according to the established ACMG criteria in a systematic way. This tool supports consistency and accuracy in variant assessment by guiding the users through consideration of evidence present and the criteria leading to the final classification.

**Figure 3:**
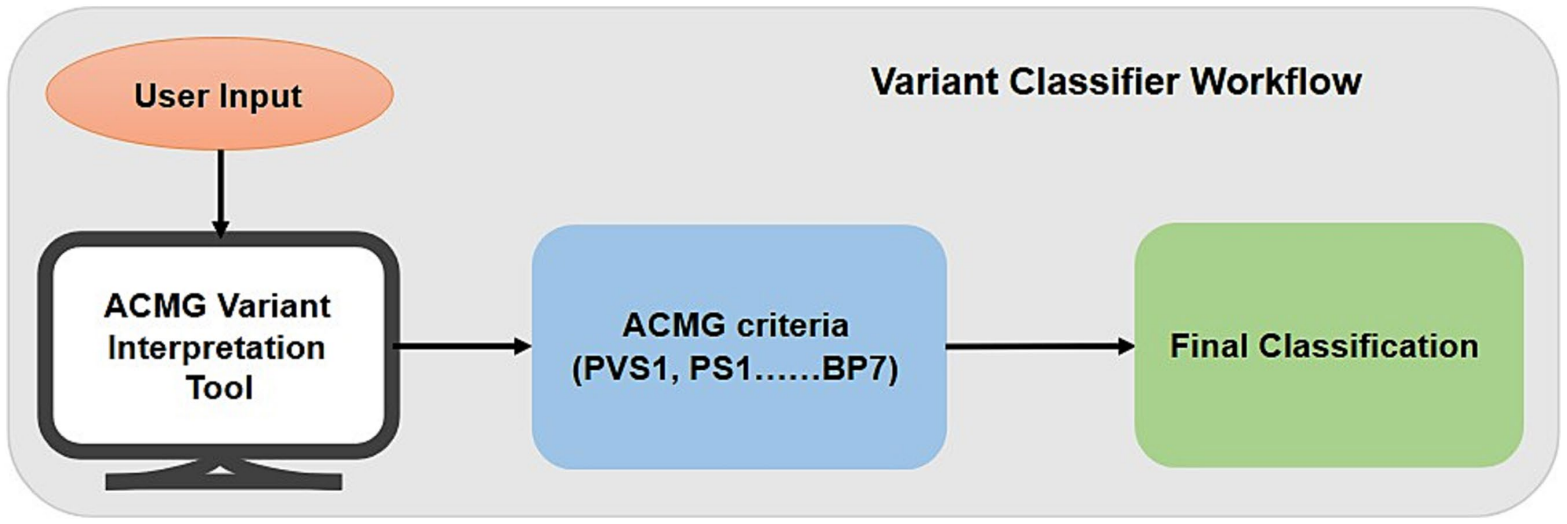
Workflow of variant classifier.

Variant classifier is a questionnaire-based web application designed for variant interpretation according to ACMG-AMP guidelines. The tool implements a hybrid decision-support system that applies the canonical ACMG combination rules followed by a series of conditional overrides and a quantitative scoring system for unresolved cases. The complete workflow of Variant classifiers is shown in Figure 3.

- Data Collection and User Interaction: The tool interactively collects the answers for the evidence through a structured questionnaire-based interface. The user is asked to supply:

- Variant and disease context: The condition associated with the variant nomenclature.
- Inheritance pattern: zygosity (homozygous, heterozygous, hemizygous) and inheritance pattern (autosomal dominant, autosomal recessive, X-linked).
- Evidence evaluation: A set of yes/no and multiple-choice questions across all ACMG evidence categories, including:

- Variant type (e.g., null, missense)
- Population frequency data
- Computational prediction data
- Functional assay results
- Segregation analysis (e.g., *de novo* status, carrier status in parents for recessive disorders)
- Cis/trans analysis
- Previous reports from reputable sources

The tool is implemented in JavaScript and follows a hierarchical decision to assign the final classification such as Pathogenic (P), Likely Pathogenic (LP), Benign (B), Likely Benign (LB) and Variant of Uncertain Significance (VUS). The classification logic follows through four sections:

- Primary classification through ACMG combination rules: The core of the tool is the application of ACMG/AMP evidence combination rules. These combination rules are based on the 28 evidence that are in the ACMG guidelines which are divided into 1 Pathogenic Very Strong (PVS), 4Pathogenic Strong (PS), 6 Pathogenic Moderate (PM), 5 Pathogenic Supporting (PP), 1 Benign Stand-Alone (BA), 4 Benign Strong (BS) and 7 Benign Supporting (BP). The user-answers are aggregated, and the algorithm checks against the predefined set of ACMG combination rules. This section outputs a classification only if any of the rules are satisfied.
- Conflict resolution: Conflict interpretations are those where evidence satisfies combination rules for both a pathogenic and a benign classification. In this case, the algorithm compares the strength of evidence from each side using the following hierarchy:

- Pathogenic evidence containing PVS or any PS criteria outweighs benign evidence and vice-versa
- Multiple PM evidence outweighs benign evidence lacking BS criteria
- BS evidence outweighs pathogenic evidence lacking PS criteria

If the strength of conflicting evidence is comparable, the variant is defaulted to VUS.

- Conditional VUS overrides for incomplete evidence: If no ACMG combination rule is satisfied, the tool checks for specific cases leading to a VUS classification. This logic section is activated only if no other strong or very strong evidence exists. The overrises check for:

- Functional studies have not been done
- The variant is not located in a known mutational hotspot or critical domain
- The variant is assumed *de novo* without confirmed parentage
- The variant is observed in healthy individuals in a genotype inconsistent with the disease
- Variant found in a case with an alternate molecular basis for disease

- Quantitative score-based classification: If the variant remains unclassified after the steps a or c, a quantitative score is calculated. Each ACMG criteria is assigned a predefined weight such as BA1 = 1, BS = 2, BP = 3 PP = 4, PM = 8, PS = 16 and, PVS1 = 32. The weights of all applied criteria are added to determine the final score. The pre-established thresholds, which are calculated based on ACMG combination rules used that defines the evidence gradient, are used to determine the final classification:

- Score towards 96: Pathogenic (P)
- Score till 48: Likely Pathogenic (LP)
- Score 17–25: VUS
- Score till 2: Likely Benign (LB)
- Score till 8: Benign (B)

The VUS range (17–25) is designed to capture variants with moderate evidence that falls short of a definitive ACMG rule combination.

Output: The tool offers a complete report containing:

- The final variant class and rationale for it (e.g., “PVS1 + PM2” or “Score-based VUS”).
- Summary count of criteria applied by evidence strength (PVS, PS, PM, PP, BA1, BS, BP).
- Quantitative total score.
- Annotated list of all activated criteria and informative notes, organized by evidence of strength.

## Graphical user interface development

The NMPhenogen database is accessible through a web-based graphical user interface (GUI) developed using HTML, CSS, and JavaScript, hosted on GitHub Pages. GUI provides a comprehensive platform for clinicians, researchers, and geneticists to interact with the NMPhenogen database, enabling seamless access to the NMPhenoscore tool including for disease prioritization and the Variant classifier for variant classification. The interface is designed to facilitate user-friendly navigation, ensuring that users can input clinical symptoms, query the genetic variants, and retrieve the results without requiring extensive technical expertise. The GUI leverages HTML5 for structured content, CSS3 for responsive and visually appealing styling, and JavaScript for dynamic interactivity. Responsive design principles ensure compatibility across devices, including desktops, tablets, and smartphones, making the tools accessible across clinical and research. JavaScript enables real-time processing of user inputs, such as symptom lists for NMPhenoscore or evidence responses for the Variant classifier, and displays outputs, such as prioritized disease lists or variant classification reports, in a clear and organized format. For instance, NMPhenoscore results are visualized using interactive virtual sunburst plots to enhance the interpretability of genotype–phenotype associations. The SQLite3 database, containing comprehensive data on 747 genes associated with neuromuscular genetic disorders, is integrated into the backend, allowing efficient querying and retrieval of gene, variant, and phenotype information.

The GUI, along with the NMPhenogen database and tools, is publicly available via the project’s GitHub Pages repository. Comprehensive documentation, including implementation details, usage instructions, and example workflows, is provided within the repository to support users in effectively utilizing the platform. This open-access approach promotes transparency, reproducibility, and collaboration in the study of neuromuscular genetic disorders.

## Results

**Figure 4:**
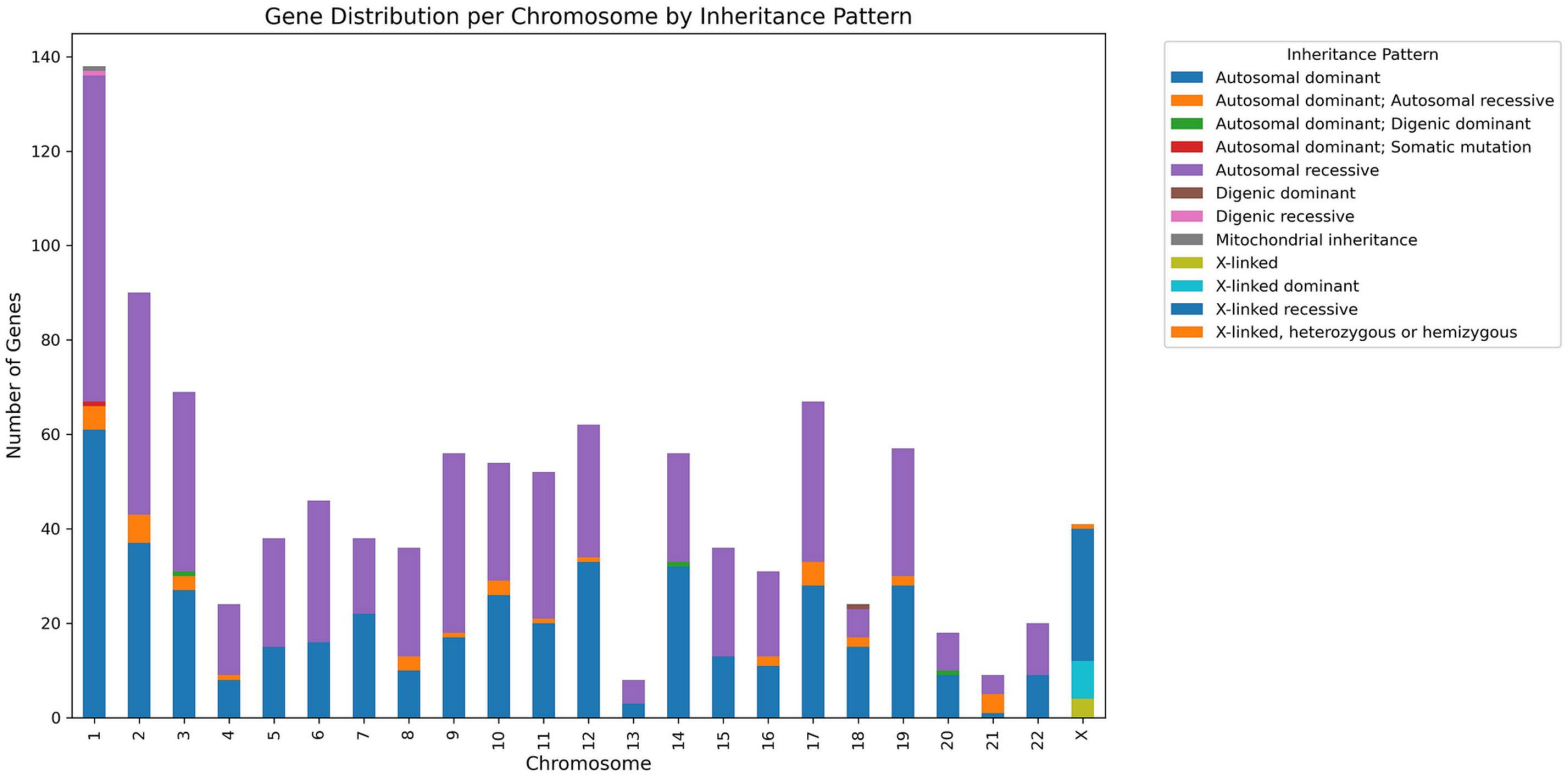
Gene distribution per chromosome by inheritance pattern.

The database has 747 genes associated with NMGDs. Figure 4 gives detailed information regarding the chromosome and gene association along with inheritance.

### The NMPhenoscore

**Figure 5:**
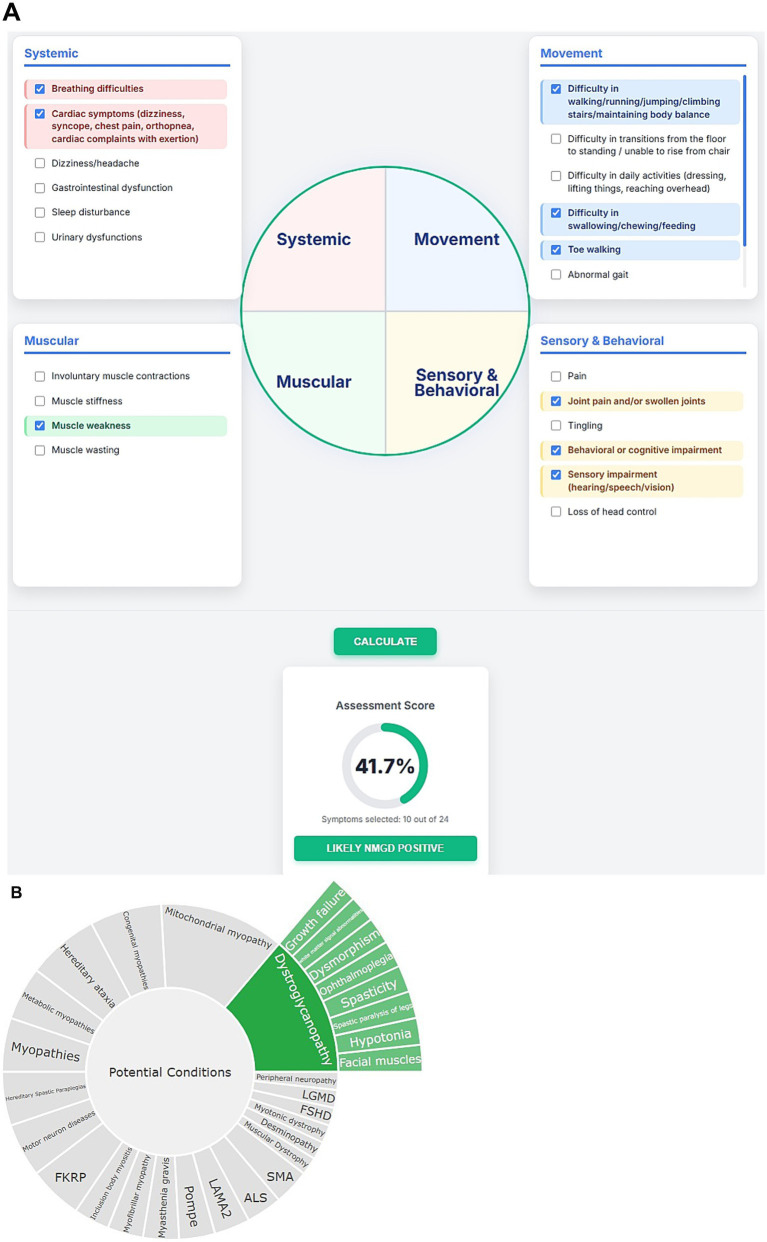
**(A)** Visual representation of initial screening for NMGD. **(B)** Sunburst visualization of gene-phenotype associations.

The performance of the NMPhenoscore tool was evaluated for initial screening for NMGD by selecting symptoms such as muscle weakness, frequent falls, breathing difficulties, cardiac involvement, difficulty in swallowing, difficulty in climbing stairs, toe walking, join contractions, cognitive impairment, and impaired vision. The assessment indicates a 41.7% score, with 10 out of 24 symptoms selected, suggesting a likely NMGD Positive result (Figure 5A). These findings point toward possible neuromuscular involvement. To evaluate the second part of the tool that is to check the specific condition of NMGD, symptoms like ophthalmoplegia, facial muscles, hypotonia, white matter signal abnormalities, dysmorphism, growth failure, spasticity and spastic paralysis of legs were selected in an unbiased manner from Human Phenotype Ontology (HPO). All these features aligned with the knowledgebase, leading the tool to recommend Dystroglycanopathy as the top condition. This prediction was concordant with HPO, where these symptoms were found to be associated with Dystroglycanopathy. Alongside, the tool will provide visualizable results in the form of a sunburst for a better understanding of associations (Figure 5B).

### Variant classifier

To evaluate the variant classifier, 100 gene variants (variants, data is given in Supplementary file 3) were taken from ClinVar in an indiscriminate manner. These variants were distributed evenly across the five classifications, namely VUS, P, LP, LB and B, with 20 variants in each group. Each variant was classified by three sources such as ClinVar (see text footnote 1), Franklin^12^ and the variant classifier to compare the final classification from all three sources. The observations are as follows:

- Out of 20 VUS variants, one variant was classified as LB in variant classifier by answering the questions based on the evidence available indicating 95% concordance with ClinVar. But, in the case of Franklin, two were classified as B, one was classified as LB and one variant was classified as LP indicating a slightly shifted distribution with 80% concordance with ClinVar. The discrepancy in Franklin classification can be attributed to misannotated null variant and incorrect citation of source (described in supplementary file 3).
- Out of 20 P and 20 LP variants, one was classified as LP and nine were classified as P, respectively in variant classifier indicating 97.5% concordance with ClinVar whereas Franklin showed six LP, two VUS and one unknown variant for P and seven P, and one unknown variant for LP indicating 90% concordance with ClinVar.
- Out of 20 B and 20 LB variants, five variants were classified as LB for B, two B and one VUS for LB in Variant classifier indicating 97.5% concordance with ClinVar whereas two were classified as LB for B and five B and one VUS for LB in Franklin indicating 97.5% concordance with ClinVar.

**Figure 6:**
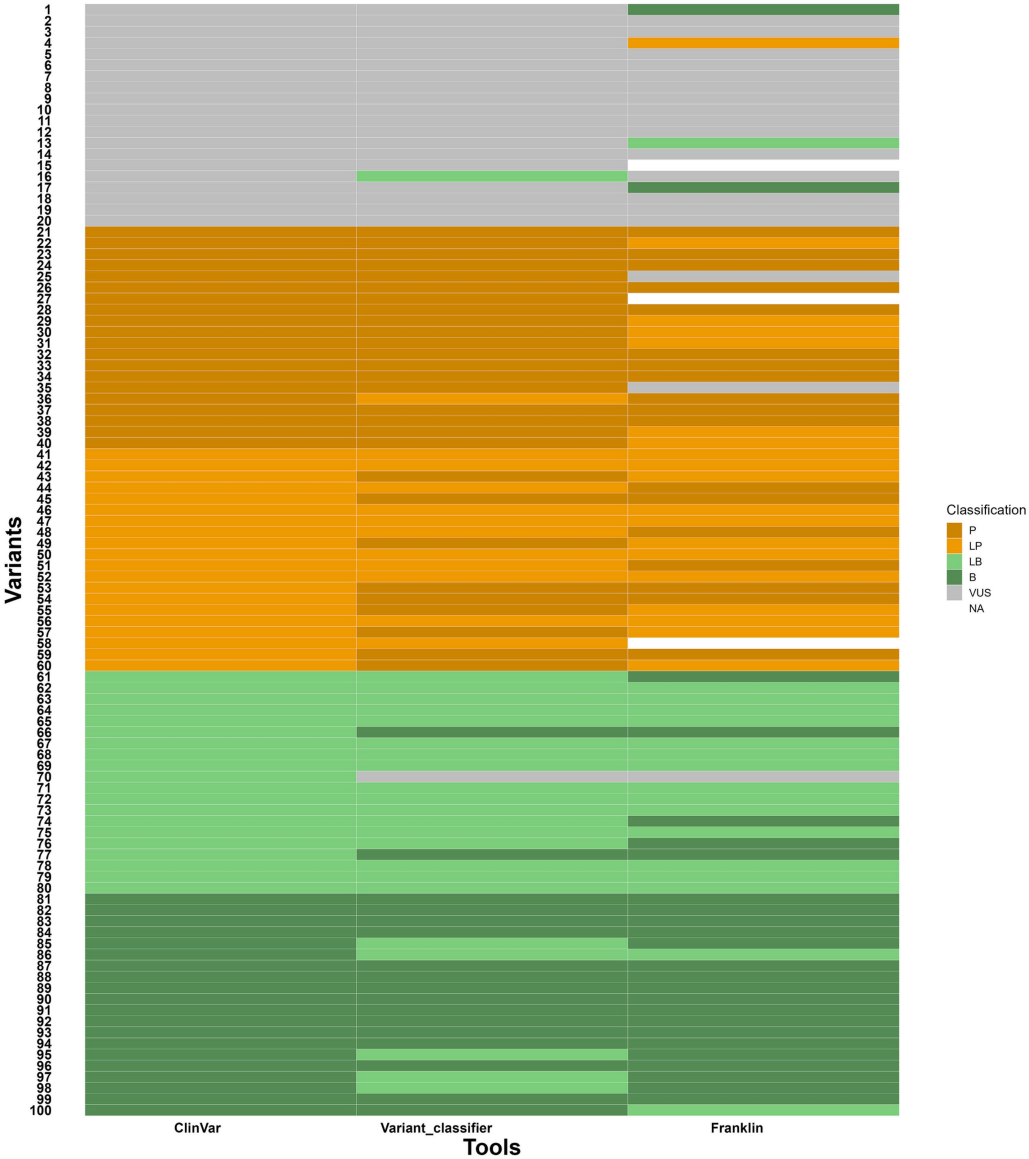
Heatmap showing comparison of variant classifications between variant classifier, ClinVar and Franklin. Y axis- variants (numbers given instead of variant ID) and X axis- tools for comparison.

With these results, the variant classifier classified variants consistently with these reference sources, though minor differences were observed in borderline cases. The comparison is given in the heatmap (Figure 6). It is important to note that ClinVar submissions are time-sensitive and classifications change as new evidence like segregation data, *de novo* results, functional assays and population frequency becomes available. Several ClinVar entries, especially older ones, may not include these data at the time of submission. Since our assessment defaulted segregation and de-novo requirements to “No,” discrepancies in the results likely reflect missing evidence that affected the classification of ClinVar and Franklin. Franklin, specifically, updates its database with more recent evidence on a regular basis, which can possibly account for observed changes versus ClinVar and our Variant classifier tool. As compared to Franklin, which prompts for phenotype association, ethnicity, zygosity statuses as optional entries, variant classifier makes the enquiries for zygosity, segregation, allele frequency, genotype–phenotype correlation, functional studies along with references/databases to gather the information and processes.

## Discussion

Neuromuscular genetic disorders are a broad group of inherited conditions marked by progressive muscle weakness, degeneration, and impaired motor function. A major challenge in diagnosing these disorders is their significant genetic diversity. Around 747 genes are associated with NMGDs and variations in different genes often lead to similar clinical symptoms. Moreover, different variants within the same gene lead to distinct traits and at the same time, similar symptoms may come from variants in unrelated genes. This heterogeneity makes the diagnosis complicated, especially when the patients show variable symptoms and age of onset. Barriers such as cost, availability, and limited awareness of NGS delay testing, particularly in resource limited settings. High neonatal mortality is common due to early respiratory failure, feeding issues, and infections in conditions like SMA type 0/I, congenital myotonic dystrophy, and mitochondrial myopathies. Early detection through newborn screening (NBS) or rapid genomic sequencing of symptomatic individuals can dramatically improve outcomes, with therapies preventing disease progression. The reliable prioritization of diseases based on phenotypic characteristics and precise interpretation of genetic variants are essential parts of diagnosis and treatment. However, these tasks are challenging due to the variability in available evidence and clinical heterogeneity observed. In our study, we developed two computational tools- one for disease prioritization (NMPhenoscore) and another for variant classification (Variant classifier) to support genetic diagnosis. The ability to intuitively correlate phenotypes to potential disease is essential for both research and clinical utility, especially in multidisciplinary contexts where non-specialists may require understandable representation of genotype–phenotype relationships. NMPhenoscore integrates manual literature curation with computational preprocessing, ensuring that both common and disease defining features are systematically captured. It is linked to HPO which allows the mapping of user-entered clinical features to standardized phenotypic terms, ensuring consistency in phenotype annotation. It evaluates the likelihood of having NMGDs in the first step based on the presence of most common clinical features. Later, the diseases are prioritized based on the algorithm that quantifies matches between user provided symptoms with curated disease symptom associations. When we evaluated NMPhenoscore with 10 common clinical features from a set of 24, associated with NMGDs, the tool calculated the likelihood of being affected with NMGD. For the second part we gave symptoms from HPO, and it accurately prioritized dystroglycanopathy as the top predicted condition. The result in the form of sunburst plots to facilitate clearer communication between computational outputs and clinicians make NMPhenoscore a useful phenotype driven prioritization tool.

The classification of genetic variants is important in clinical genomics, as it determines if a sequence change is pathogenic, benign or of unknown significance. Accurate classification is essential for guiding patient management, including diagnosis, prognosis, reproductive advice, and treatment options. Misclassification can lead to improper clinical handling resulting in unnecessary procedures for benign variants or missed treatment opportunities and monitoring for pathogenic variants. The variant classifier developed in our study considers these sensitive points. We compared 100 variants in an unbiased manner to evaluate the performance of the Variant classifier against ClinVar and Franklin. The results were in high concordance with ClinVar, particularly for pathogenic and benign variants (97.5 and 97.5%), and slightly lower concordance for VUS (95%) variants. However, with Franklin, the variant classification was observed to be slightly shifted due to its more conservative thresholds, resulting in more variants falling under pathogenic or benign categories. The findings from our study highlight that the Variant classifier shows strong concordance for variants at the extremes of classification (Benign and Pathogenic), but the borderline categories (LB, VUS, and LP) are prone to reclassification. This is expected because the distinction between definitive and likely mainly depends on the strength and weight of supporting evidence considered while classifying. The differences observed between our tool, ClinVar and Franklin suggest that variant classification tools may vary in sensitivity to specific types of evidence. For example, the classification of some LP variants to P by our tool reflects a stricter interpretation of strong/ very strong criteria. Similarly, the slight shift in VUS towards LB or B indicates that uncertain variants may often be benign but lack sufficient evidence to support. These observations align with earlier reports that variant classification is not fully reproducible across different labs, especially for the gray zones (LB/LP). It is important to note that the discrepancies in our results do not involve major reclassifications, supporting the overall reliability of the tool, except for eight variants that exhibited significant changes in their classification (highlighted in yellow in supplementary file 3). This underscores the need for manual curation and expert judgement for accurate and meaningful variant interpretation, especially for clinically actionable variants. Accurate reclassification of variants, especially VUS, LB, and LP, require functional and clinical validation. Experimental assays, segregation studies, and longitudinal clinical follow up are necessary to determine the biological impact of variants and confirm their association with specific disease phenotypes. In the absence of such evidence, the risk of misclassification increases adversely affecting the patient. Therefore, integrating computational predictions with functional data and clinical observations is essential for clinically meaningful variant classification. A relatively small number of variants sampled and focus on a single disease limits the evaluation of the tool. However, our tool is user defined and therefore classifies the variants based on the data provided by the user (e.g., research findings, functional analysis, supporting evidence). This offers great potential for accurate classification and reclassification of the variants. While most variant classifying tools rely on predefined algorithms or submissions from researchers as evidence for scoring. For example, ClinVar assigns a star system to the submitted variant interpretations to indicate the level of review and consensus supporting each classification. This system is largely subjective and unsupervised, as it reflects the extent of agreement among submitters rather than independent validation. For instance, a single submission from a recognized laboratory may still receive a star, while conflicting interpretations from multiple submitters get lower ratings regardless of the strength of underlying evidence. Thus, ClinVar can be a preliminary guide but cannot be considered as an absolute indicator of variant pathogenicity as ACMG classification is not mandatory to submit variants to Clinvar. In comparison with Franklin, our tool demonstrates a more evidence-driven approach by combining multiple layers of evidence such as functional, computational and clinical data for precise classification. For instance, Franklin interprets a VUS variant NM_007289.4(MME):c.-10-1G > T as LP, by considering it as null variant(PVS1). However, our tool and ClinVar classify this variant as VUS due to no evidence for the variant of being null variant as RNA/functional studies are absent. Similarly, the VUS variants NM_001127222.2(CACNA1A):c.7266_7271del (p.Ser2423_Gly2424del) and NM_053025.4(MYLK):c.*1,302 T > C is interpreted as B by Franklin based on gnomAD allele frequency (BA1 and BS2), although the reported frequency does not align with gnomAD data. Likewise, NM_000335.5(SCN5A):c.6045G > A (p.Val2015=) is classified as LB by Franklin based on the evidences BP6, BP7 and PM4 but there is no evidence supporting BP6. Another example is NM_022072.5(NSUN3):c.123-615_466 + 2155del, which is classified as VUS by Franklin despite the presence of the strong pathogenic evidences (PS3, PS4, PM1, PM4, PP3, PP4, PP5). Likewise, NM_003280.3(TNNC1):c.12C > G (p.Ile4Met) is classified as VUS by Franklin even though it meets multiple pathogenic evidences (PS1, PS3, PS4, PP2, PP3, PP4, PP5). These discrepancies highlight the strength of the Variant Classifier, which systematically applies ACMG guidelines and considers the available evidence. This results in more accurate and clinically relevant classifications, particularly in cases where borderline or conflicting interpretations exist.

To conclude, NMPhenoscore aids in narrowing down the candidate diseases from clinical features and Variant classifier systematically classifies underlying genetic alterations. Integration of both approaches will substantially improve diagnostic yield, particularly in complex conditions where either phenotype or genotype information alone is not sufficient. However, broader validation using larger and more diverse phenotype sets and systematic comparison with established platforms is required to determine the robustness of NMPhenoscore in diagnostic workflows. Expanding the knowledgebase to include additional clinical features and conditions and refining the scoring algorithm to capture symptom specificity and progression will strengthen its performance. The Variant classifier also requires larger studies across diverse genes and variants associated with different disease conditions to assess its generalizability beyond neuromuscular disorders. Integration of emerging genomic and functional evidence, as well as population specific data, along with enhancing interoperability with existing genomic resources represents a key future direction for both tools to improve classification accuracy. The combined use of both the tools validated through larger clinical datasets and real-world case studies, has the potential to streamline diagnostics and contribute to more accurate, consistent, and meaningful patient care. We invite researchers, genetic counselors, clinical geneticists, clinicians and genome analysts to use our database and tools in their genotype–phenotype correlation activity and give us feedback to improve the accuracy of the database.

## Data Availability

The original contributions presented in the study are included in the article/Supplementary material, further inquiries can be directed to the corresponding author.

## Footnotes

^1^ https://www.ncbi.nlm.nih.gov/clinvar/

^2^ https://varsome.com/

^3^ https://franklin.genoox.com/clinical-db/home

^4^ https://www.lovd.nl/

^5^ https://wintervar.wglab.org/

^6^ https://genebe.net/

^7^ https://www.omim.org/

^8^ https://www.orpha.net/

^9^ https://www.malacards.org/

^10^ https://www.ncbi.nlm.nih.gov/search/

^11^ https://asia.ensembl.org/Homo_sapiens/Info/Index

^12^ https://franklin.genoox.com/clinical-db/home

## Supplementary material

SUPPLEMENTARY FIGURE 1 Chord diagram for NMGD knowledgebase with symptoms and conditions

SUPPLEMENTARY FILE 1 Excel sheet with list of 747 genes (nuclear and mitochondrial) associated with NMGD

SUPPLEMENTARY FILE 2 Excel sheet with list of 141 symptoms and 34 conditions (sheet 1) and potential clinical features (sheet 2) for NMGDs

SUPPLEMENTARY FILE 3 Excel sheet with list of variants used for evaluating the Variant classifier

## Glossary

NMGDs: Neuromuscular genetic disorders
LGMD: Limb Girdle Muscular Dystrophy
DMD: Duchenne Muscular Dystrophy
DM1: Myotonic Dystrophy 1
MERRF: Myoclonic epilepsy with ragged red fibers
MELAS: Mitochondrial encephalopathy, lactic acidosis and stroke like episodes
CMDs: Congenital Muscular Dystrophy
FSHD: Facioscapulohumeral muscular Dystrophy
CPK: Creatinine Phosphokinase
CNS: Central Nervous System
HSPs: Hereditary Spastic Paraplegias
SMA: Spinal Muscular Atrophy
NGS: Next Generation Sequencing
ACMG: American College of Medical Genetics
AMP: Association for Molecular Pathology
CAP: College of American Pathologists
OMIM: Online Mendelian Inheritance in Man
VUS: Variant of Uncertain Significance
HPO: Human Phenotype Ontology
OMIM: Online Mendelian Inheritance in Man
MIM: Mendelian Inheritance in Man
EMG- Electromyography
MRI: Magnetic Resonance Imaging
CDSS: Clinical Decision Support System
