# Supplementary figures and images for: NMPhenogen: a comprehensive database for genotype–phenotype correlation in neuromuscular genetic disorders
Source: Front Neurosci. 2025 Nov 4;19:1696899. doi: 10.3389/fnins.2025.1696899 (PMC12623376; doi:10.3389/fnins.2025.1696899)

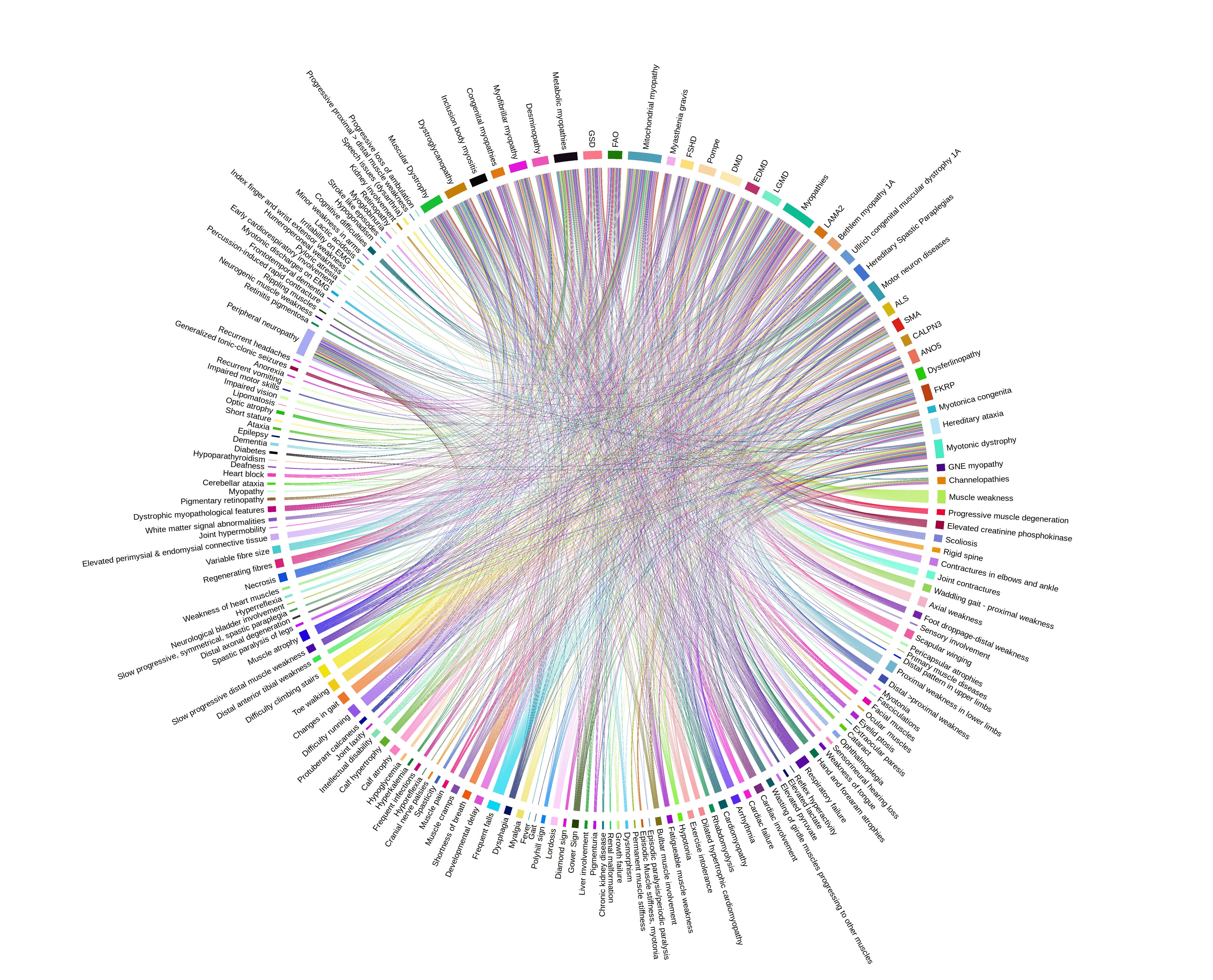

Supplement: SUPPLEMENTARY FIGURE 1 — Chord diagram for NMGD knowledgebase with symptoms and conditions [file Image_1.JPEG]
